# Retraction Note: Ranking candidate genes of esophageal squamous cell carcinomas based on differentially expressed genes and the topological properties of the co-expression network

**DOI:** 10.1186/s40001-015-0130-8

**Published:** 2015-03-26

**Authors:** Yuzhou Shen, Jicheng Tantai, Heng Zhao

**Affiliations:** Department of Thoracic Surgery, Shanghai Chest Hospital, Shanghai Jiao Tong University, 241 Huaihai (West) Rd, Shanghai, 200030 People’s Republic of China

## Retraction

The Publisher and Editor regretfully retract this article [1] because the peer-review process was inappropriately influenced and compromised. As a result, the scientific integrity of the article cannot be guaranteed. A systematic and detailed investigation suggests that a third party was involved in supplying fabricated details of potential peer reviewers for a large number of manuscripts submitted to different journals. In accordance with recommendations from COPE we have retracted all affected published articles, including this one. It was not possible to determine beyond doubt that the authors of this particular article were aware of any third party attempts to manipulate peer review of their manuscript.

## References

[1] Shen Y, Tantai J, Zhao H (2014). Ranking candidate genes of esophageal squamous cell carcinomas based on differentially expressed genes and the topological properties of the co-expression network. Eur J Med Res.

